# Amygdala structure and function and its associations with social-emotional outcomes in a low-risk preterm sample

**DOI:** 10.1007/s00429-023-02749-1

**Published:** 2024-01-18

**Authors:** L. Fernández de Gamarra-Oca, O. Lucas-Jiménez, J. M. Ontañón, B. Loureiro-Gonzalez, J. Peña, N. Ibarretxe-Bilbao, M. A. García-Guerrero, N. Ojeda, L. Zubiaurre-Elorza

**Affiliations:** 1 Department of Psychology, Faculty of Health Sciences, University of Deusto, Bilbao, Bizkaia Spain; 2 OSATEK, MR Unit, Galdakao Hospital, Galdakao, Spain; 3 Division of Neonatology, Biocruces Health Research Institute, Cruces University Hospital, Barakaldo, Bizkaia Spain

**Keywords:** Low-risk preterm birth, Functional connectivity, Amygdala, Social-emotional outcomes, Young adulthood

## Abstract

Amygdala atypical volume development and functional connectivity (FC) at small gestational ages (GA) have been found across childhood. This adult-oriented study assesses whether altered amygdala structure and function is present following low-risk preterm birth. T1-weighted and resting-state functional MRI images of 33 low-risk preterm (30–36 weeks’ GA) and 29 full-term (37–42 weeks’ GA) young adults of both sexes, aged between 20 and 32 years old, were analyzed using FreeSurfer (v6.0.0) and Coon Toolbox (v21.a). The social-emotional assessment included Happé’s Strange Stories Test, the Moral Judgment Test, Delay-Discounting Test, Adult Self Report, and Emotion Regulation Questionnaire. No differences were found in social-emotional outcomes or amygdala volumes between the groups. Low-risk preterm young adults showed increased FC between the left amygdala, right amygdala and medial frontal cortex (MedFC) (*F* = 9.89, *p*-FWE = 0.009) at cluster level compared to their full-term peers. However, significant results at connection level were not observed between left and right amygdala. Lastly, increased FC at cluster level between the right amygdala and MedFC, and left amygdala and MedFC, was related to better social-emotional outcomes only in low-risk preterm young adults (*F* = 6.60, *p*-FWE = 0.036) at cluster level. At connection level, in contrast, only right amygdala–MedFC increased FC was significantly associated with better social-emotional outcomes. This study reveals that low-risk prematurity does not have an effect on social-emotional outcomes or structural amygdala volumes during young adulthood. However, individuals who were considered to be at a lower risk of exhibiting neurodevelopmental alterations following preterm birth demonstrated increased FC between the left and right amygdala and MedFC.

Prematurity is defined as any birth before 37 complete weeks of gestation (WHO 1997). Neonates suffering from subtle neurological abnormalities and perinatal comorbidities who were born low-risk preterm [30- to 36-week gestational age (GA)] make up the majority of preterm deliveries (Caravale et al. 2005; Hart et al. 2008). Moreover, most children born low-risk preterm exhibit a profile of cognitive and behavioral problems shared with children born at term predominantly with socioeconomic risk factors (Johnson et al. 2018). However, brain alterations following low-risk preterm birth may be the cause of their plausibly unfavorable long-term neurodevelopment (Walsh et al. 2014). In line with this, social-emotional functioning appears to be essential for optimal long-term neurodevelopment (Oberle et al. 2014; Peralta-Carcelen et al. 2018), especially since children and adolescents born moderate and late preterm are at higher risk of emotional and behavioral problems (Cheong and Doyle 2020).

Emotional symptoms (e.g., often unhappy, downhearted, etc.) have been predicted by different brain volumetric values following very preterm birth (< 32-week GA), including amygdala structure (Liverani et al. 2023). The amygdala is highly innervated by glucocorticoid receptors, and the peak of corticotropin-releasing hormone receptor density occurs during the early postnatal weeks, making the newborn stage a time of increased susceptibility to stress (VanTieghem and Tottenham 2018). Following preterm delivery, structural alterations in the amygdala have been found at term equivalent age and childhood (Cismaru et al. 2016; Mueller et al. 2022). Moreover, the presence of altered amygdala functional connectivity (FC) following very preterm birth suggests that there is a different pattern of associations with social-emotional abilities (e.g., emotion regulation) compared to those born at term, which may point to a reduced degree of functional architecture maturation (Kanel et al. 2022; Rogers et al. 2017; Siffredi et al. 2022). According to a study based on extremely preterm neonates, prenatal stress leads to a significantly reduced connection between the left amygdala and other areas when compared to those not suffering from such stress (Scheinost et al. 2016).

The amygdala–prefrontal cortex circuit is also impacted by negative experiences and stress (VanTieghem and Tottenham 2018). In the general population, early life stress has been linked to increased negative centromedial amygdala–ventromedial prefrontal cortex connection (Miller et al. 2021). Changes in the function of this circuit seem to have an effect on how emotions are regulated and increase the risk of psychiatric disorders (VanTieghem and Tottenham 2018). For instance, greater exposure to pain-related stress in infants born low-risk preterm has been linked to disrupted developmental scores (Morag et al. 2017).

From a neurodevelopmental perspective, individuals’ reactions to emotional events shift significantly from childhood to adulthood (Silvers et al. 2017). The amygdala–prefrontal brain circuit continues developing until early adulthood despite the fact that amygdala reactivity diminishes during aging (VanTieghem and Tottenham 2018). However, in preterm populations, although decreased amygdala volume and altered FC have been described, only social functioning (e.g., increased social difficulties, heightened levels of anxiety and depression, reduced extroversion, and poor self-esteem) correlates with altered amygdala–posterior cingulate FC, with no structural associations shown during adulthood (Johns et al. 2019; Schmitz-Koep et al. 2021a). In fact, due to functional changes in key emotion processing brain networks, including suboptimal amygdala modulation, adults born very preterm continue to exhibit subtle abnormalities in emotion recognition (Papini et al. 2016). Nevertheless, there are still no data available concerning low-risk preterm samples.

Deficits of social-emotional abilities in those born preterm have a substantial influence on their quality of life and on the burden of healthcare in society (Linsell et al. 2019; Raju et al. 2017). Furthermore, atypical development of the amygdala subnuclei volumes together with its altered FC have been related to social-emotional abilities in children born very preterm (Mueller et al. 2022). For this reason, and given the lack of studies that focus on those considered to be at lower risk of exhibiting neurodevelopmental alterations following preterm birth, this study aims to assess the different social-emotional profiles between young adults born low-risk preterm and their full-term counterparts. Besides, it aims to analyze amygdala structure and its FC with medial frontal cortex (MedFC) in young adults born low-risk preterm. Finally, the study seeks to evaluate the possible associations during young adulthood between social-emotional outcomes and amygdala volumes and FC (i.e., amygdala–MedFC) following low-risk preterm birth. It was hypothesized that, based on the literature (Cheong and Doyle 2020; Cismaru et al. 2016; Mueller et al. 2022), young adults born low-risk preterm would have reduced amygdala volumes and FC, as well as obtaining lower social-emotional scores, than the full-term controls.

## Methods

### Participants

Sixty-two young adults in total (aged between 20 and 32 years) took part in this prospective observational cross-sectional study: 33 low-risk preterm (M_age_ = 25.33 years; SD_age_ = 2.92) and 29 full-term (M_age_ = 26.41 years; SD_age_ = 2.24) subjects. A group of preterm young adults at lower risk of exhibiting major disabilities was recruited at Cruces University Hospital (Barakaldo, Spain) by chain-referral sampling from September 2018 to April 2021. This group of 33 young adults born preterm at 30–36 weeks of gestation and with the following inclusion/exclusion criteria were considered to be at lower risk of adverse neurodevelopmental outcomes as previously reviewed by our research group (Fernández de Gamarra-Oca et al. 2021): (1) 30- to 36-week gestational age (GA), (2) absence of brain pathology identified by neonatal cranial ultrasound, (3) lack of substantial neonatal morbidity (i.e., congenital neurological, cardiac, respiratory or digestive malformations, necrotizing enterocolitis, or septic shock), and (4) ranging in age at time of evaluation from 20 to 40 years. A full-term group of 29 subjects was recruited, with the inclusion criteria for these participants being as follows: (1) > 37-week GA, and (2) ranging in age at time of evaluation from 20 to 40 years. The exclusion criteria for both low-risk preterm and full-term groups were a history of acquired brain injury, cerebral palsy or any other neurological impairment, congenital malformations, and chromosomal abnormalities.

### Measures

#### Social-emotional assessment

Social-emotional functioning was measured according to the following domains, with the instruments used to measure each domain being described below. All instrument scores (i.e., theory of mind and moral competence total scores, delay gratification ratio, internalizing and externalizing problem scores and cognitive reappraisal and expressive suppression punctuations) were standardized by transforming them into *z*-scores to create a composite score to measure social-emotional functioning (henceforth referred to as social-emotional outcomes). Cronbach’s alpha (reliability) coefficient was 0.78.

*Theory of mind* was measured using the Happé’s Strange Stories Test (Happé 1994) by selecting four of the original stories to represent a lie, white lie, misunderstanding, and pretense. The participant is asked to deduce the thoughts and intentions of the characters in each narrative. For example, in the mentalizing story involving a double bluff, a 2-point answer might be the prisoner tried to trick the army by telling the truth, and so the army will look on the opposite side to the one he says. A 1-point answer for the same story would demonstrate partial understanding: reference the results or a simple bluff. A 0-point answer would be incorrect, such as since the prisoner was afraid, he wanted to reveal the truth.

*Moral competence* was evaluated by means of the Moral Judgment Test (MJT) (Lind 1978), characterizing the ability to make morally upstanding decisions and judgments in two dilemma situations—the “workers’ dilemma” (i.e., refers to labor rights and professional ethics) and “mercy killing dilemma” (i.e., refers to euthanasia in a medical setting). The MJT specifically assesses the respondent’s ability to score arguments (for and against a protagonist’s action in a dilemma situation) in terms of moral quality rather than opinion agreement. For most participants, this is a tough task; only a few responders score greater than 40 on the C-scale from 0 to 100.

The Delay-Discounting Test (DDT) (Kirby and Maraković 1996), also called the Monetary Choice Questionnaire, assessed *delayed gratification* by participants answering 21 questions, such as the following: “Would you rather have €85 in 14 days or €30 now?”.

The Adult Self Report (ASR) was employed as a measure of *emotional-behavioral functioning* (Achenbach et al. 2003). The questionnaire provides scores for the following scales: anxious/depressed, withdrawn, somatic complaints, thought problems, attention problems, aggressive behavior, rule-breaking behavior, and intrusive behavior. Internalizing and externalizing problems were also obtained from the sum of these scales.

The 10-item Emotion Regulation Questionnaire (ERQ) measures participant predisposition for both *cognitive reappraisal* and *expressive suppression* of their emotions (Gross and John 2003). Participants respond to each item on a Likert-type scale, ranging from 1 (strongly disagree) to 7 (strongly agree).

#### General cognitive assessment

The Raven’s Advanced Progressive Matrices (RPM) test was used to assess *analogical reasoning* as general non-verbal cognition (Raven and Court 1998), while the Peabody Picture Vocabulary Test III (PPVT-III) was employed to measure *receptive language,* since it characterizes general verbal cognitive performance (Dunn and Dunn 1981).

#### Socioeconomic status (SES)

The occupation and educational domains of participants and their parents were taken into account separately using the Hollingshead Index (Hollingshead 2011). The participants’ *self-SES* (i.e., participants’ ongoing occupation and highest level of education) and *familial SES* (i.e., the average of parents’ retrospective occupation and highest level of education) were thus two distinct measures that were obtained.

### Image acquisition

3-dimensional MRI datasets were obtained from the Magnetic Resonance Imaging Unit OSATEK, Galdakao Hospital (Galdakao, Spain) for 64 participants; however, two were eventually excluded due to difficulties in the acquisition process. The radiologist (J.M.O.) reported that all MRI images were non-pathological. Hence, there was a final sample of 33 low-risk preterm and 29 full-term young adults. A single session was used to acquire both sequences. T1-weighted images were also obtained from a Philips 3T Achieva dStream (axial orientation, 3 min 58.9 s session, 160 slices, TR/TE = 8.1/3.7 ms, 288 × 248 matrix, 8° flip angle, 1 mm slice thickness, no gap), and resting-state functional MRI (rs-fMRI) was also acquired: 8 min session, 315 whole-brain gradient echo-planar images, 44 axial slices, TR/TE = 1500/30 ms, FOV = 240 × 240 × 132 mm^2^, and voxel size = 3 × 3 × 3 mm^3^.

Before acquiring rs-fMRI, the participant’s head was restrained from movement and protected from scanner noise using foam padding and earplugs. Subjects were told not to perform any specific cognitive or physical tasks, keep their eyes closed, and avoid falling asleep. After scanning, participants completed the Amsterdam Resting-State Questionnaire (ARSQ) (Alexander Diaz et al. 2013), which is a 27-item self-report survey covering seven dimensions of *resting-state cognition* (i.e., discontinuity of mind, theory of mind, self, planning, sleepiness, comfort, and somatic awareness).

### Image preprocessing

#### Whole and subnuclei amygdala volumes

FreeSurfer (https://surfer.nmr.mgh.harvard.edu/) (version v6.0.0) was used in order to obtain whole and subnuclei amygdala volumes from 3-dimensional T1-weighted MRI scans (Fischl 2012). T1-weighted image processing included several procedures: intensity non-uniformity correction, skull stripping, affine transformation to an MNI template, intensity normalization, removal of non-brain tissue, linear and non-linear transformations to a probabilistic brain atlas, and the labeling of subcortical/allocortical structures. Spatial localization priors were used to determine the correct label voxel-per-voxel, and skull stripping, intensity normalization, white matter segmentation, and surface extraction errors were all manually corrected where necessary. Subsequently, the amygdala was segmented automatically based on the Saygin et al. (2017) atlas into central, lateral, basal, accessory basal, cortical, medial, and paralaminar nuclei, as well as into the corticoamygdaloid transition area and anterior amygdaloid area.

#### Resting-state functional connectivity (FC)

FC analyses were carried out using the CONN Functional Connectivity Toolbox version 21.a (Whitfield-Gabrieli and Nieto-Castanon 2012). Each subject’s 315 functional images were realigned and unwarped, their slice-timing corrected, coregistered with structural data, spatially normalized into standard MNI space (Montreal Neurological Institute, Canada), and their outliers detected (ART-based scrubbing; to eliminate any influence of outlier scans on the BOLD signal, noise components are employed as potential confounding effects). During outlier detection (intermediate level, 97th percentile: 0.9 mm 5 s.d.), potential outlier scans of frame-wise displacements > 0.9 mm or BOLD signal changes > 5 s.d. were detected and flagged as potential outliers. Finally, they were smoothed with an 8-mm Gaussian kernel full with half maximum (FWHM) to increase the signal-to-noise ratio. No outliers were detected in this sample. The default preprocessing pipeline for volume-based analysis (to MNI space) was used for all preprocessing steps, and the structural data were normalized and segmented into GM, WM, and CSF. The anatomical CompCor method was used to reduce noise by extracting the principal components (5 each) from the WM and CSF time series. The subject’s estimated motion parameters and other artificial effects, such as BOLD signals in WM and CSF, which were included as additional confounds, were then removed by a denoising process that was implemented using linear regression and band-pass filtering (0.008–0.09 Hz) (Weissenbacher et al. 2009).

### Statistical and imaging analysis

Normal distribution of data was assessed using the Kolmogorov–Smirnov test (K–S), while the Mann–Whitney U test was used to analyze differences in GA, and self- and familial SES. Furthermore, the Chi-squared test was required to assess differences in two qualitative sociodemographic characteristics: gender and handedness. Student’s *t*-tests were also performed to compare birthweight (BW) and age at time of evaluation between the groups.

A multivariate analysis of covariance was conducted to compare different social-emotional domains (adjusted for age and self-SES), whole amygdala volumes (adjusted for age, self-SES, and eTIV), and amygdala subnuclei volumes (adjusted for age, self-SES, and left/right amygdala volume), while partial eta squared was employed to calculate effect sizes; around 0.01 is considered a small size effect, 0.06 medium, and above 0.14 is considered large. Moreover, bivariate correlation analyses were performed to assess the relationships between social-emotional outcomes and amygdala volumes (i.e., whole and subnuclei volumetric values). For all preceding analyses, the SPSS version 28 was used, and significance level set at 0.05.

Regarding FC statistical analysis, cluster-level inferences in region of interest (ROI)-to-ROI analyses based on Threshold Free Cluster Enhancement (TFCE) approach was used (Smith and Nichols 2009). This approach is used for thresholding ROI-to-ROI parametric maps while appropriately controlling the family-wise error (FWE) rate using TFCE analyses with a FWE corrected *p* < 0.05 at cluster level, with the post hoc test FDR corrected *p* < 0.05 at connection level. Between-group differences were assessed (with age and self-SES used as covariates) with Cluster-level ROI-to-ROI based on TFCE and the ROIs selected based on the Harvard–Oxford Structures Atlas (http://fsl.fmrib.ox.ac.uk/fsl/fslwiki/Atlases) were as follows: (a) left amygdala, (b) right amygdala, and (c) MedFC. In addition, Pearson correlation analyses (i.e., each group independently) between the strength of FC and social-emotional data were also assessed with the same approach (Cluster-level ROI-to-ROI based on TFCE) and with the selected ROIs.

## Results

Neonatal, sociodemographic, cognitive variables, and rs-fMRI mean movement are shown in detail in Table 1. As expected, there were significant differences in neonatal variables (GA and BW) between the groups. No differences were found in sociodemographic (i.e., sex, age, handedness, and familial SES), general cognitive measures (i.e., receptive language and analogical reasoning) or rs-fMRI mean movement between the groups, except in the case of the self-SES, with the low-risk preterm sample reporting lower scores. The following analyses will therefore be adjusted for self-SES together with specific covariates (see “Methods”, Statistical analysis).

**Table 1:** Neonatal, sociodemographic, and cognitive data

	Low-risk preterm *n* = 33 mean ± SD	Full-term *n* = 29 mean ± SD	Statistics (*p*)
**Neonatal data**			
GA (wks) [range]	34.48 ± 1.50 [30–36]	39.55 ± 0.88 [38–42]	***U* = 957.00 (< 0.001)**
Preterm birth type (very preterm/moderate preterm/late preterm)	1/10/22	–	
BW (g)	2158.10 ± 345.14	3298.13 ± 427.74	***t* = −11.607 (< 0.001)**
**Sociodemographic data**			
Sex (male/female)	15/18	14/15	*X*^2^ = 0.05 (0.82)
Age (yrs) [range]	25.33 ± 2.92 [20–30]	26.41 ± 2.24 [21–32]	*t* = −1.643 (0.11)
Handedness (right-handed/left-handed)	30/3	26/3	*X*^2^ = 0.03 (0.87)
Self-SES	38.36 ± 14.68	49.93 ± 12.49	***U* = 688.50 (0.003)**
Familial SES	42.06 ± 14.92	37.07 ± 13.80	*U* = 370.50 (0.13)
**Cognitive data**			
PPVT-III, receptive language	167.21 ± 13.22	167.34 ± 8.95	*U* = 420.50 (0.41)
RPM, analogical reasoning	24.15 ± 5.73	24.48 ± 4.28	*t* = −0.26 (0.80)
**rs-fMRI data**			
Mean motion	0.107 ± 0.034	0.116 ± 0.042	*t* = −0.991 (0.33)

Significant results are shown in bold

Note: *SD* standard deviation, *GA* gestational age, *wks* weeks, *BW* birthweight, *g* grams, *yrs* years, *U* Mann–Whitney U test, *t* Student *t*-test, *X*^*2*^ Chi-square test, *SES* socioeconomic status, *PPVT-III* Peabody Picture Vocabulary Test III, *RPM* Raven’s Advanced Progressive Matrices, *rs-fMRI* resting-state functional MRI

### Aim 1: differences in social-emotional outcomes

In terms of social-emotional functioning, as shown in Table 2, no differences were shown between young adults born low-risk preterm and full-term. Neither were differences found between the groups in the composite score and social-emotional domains.

**Table 2:** Differences in social-emotional outcomes between low-risk preterm and full-term young adults

	Low-risk preterm *n* = 33 mean ± SD	Full-term *n* = 29 mean ± SD	*F*-snedecor statistic (*p*)	${\eta }_{p}^{2}$	
**Social-emotional outcomes** (adjusted for age and self-SES)					
Happé’s Strange Stories Test	6.59 ± 1.37	6.71 ± 1.27	0.90 (0.77)	0.00	
MJT	13.60 ± 7.81	16.37 ± 9.67	1.27 (0.27)	0.02	
Kirby DDT	0.66 ± 0.26	0.71 ± 0.24	0.43 (0.52)	0.01	
*Adult Self Report (ASR)*					
Anxious depressed	9.74 ± 6.12	9.19 ± 3.94	0.14 (0.71)	0.00	
Withdrawn	2.77 ± 2.97	3.05 ± 2.14	0.15 (0.70)	0.00	
Somatic complaints	3.18 ± 3.19	2.39 ± 2.80	0.86 (0.36)	0.02	
Though problems	2.08 ± 1.82	2.26 ± 1.88	0.12 (0.73)	0.00	
Attention problems	6.36 ± 4.60	7.93 ± 3.90	1.79 (0.19)	0.03	
Aggressive behavior	4.32 ± 3.39	5.18 ± 4.33	0.63 (0.43)	0.01	
Rule-breaking behavior	2.07 ± 2.10	2.23 ± 2.84	0.05 (0.82)	0.00	
Intrusive behavior	1.95 ± 1.64	1.91 ± 1.89	0.01 (0.94)	0.00	
Internalizing problems	15.69 ± 10.77	14.67 ± 7.35	0.15 (0.70)	0.00	
Externalizing problems	8.36 ± 5.67	9.49 ± 7.07	0.41 (0.53)	0.01	
*Emotion Regulation Questionnaire (ERQ)*					
Cognitive reappraisal	30.02 ± 5.96	28.99 ± 4.68	0.48 (0.49)	0.01	
Expressive suppression	12.39 ± 4.84	14.28 ± 4.77	1.96 (0.17)	0.03	
**Composite score **(adjusted for age and self-SES)					
Social-emotional outcomes	0.04 ± 0.53	−0.04 ± 0.50	0.00 (0.95)	0.00	

Note: *SD* standard deviation, *SES* socioeconomic status, *MJT* moral judgement test, *DDT* delay-discounting task, ${\eta }_{p}^{2}$ partial eta squared

### Aim 2: differences in whole and subnuclei amygdala volumes and resting-state functional connectivity (FC)

**Fig. 1:**
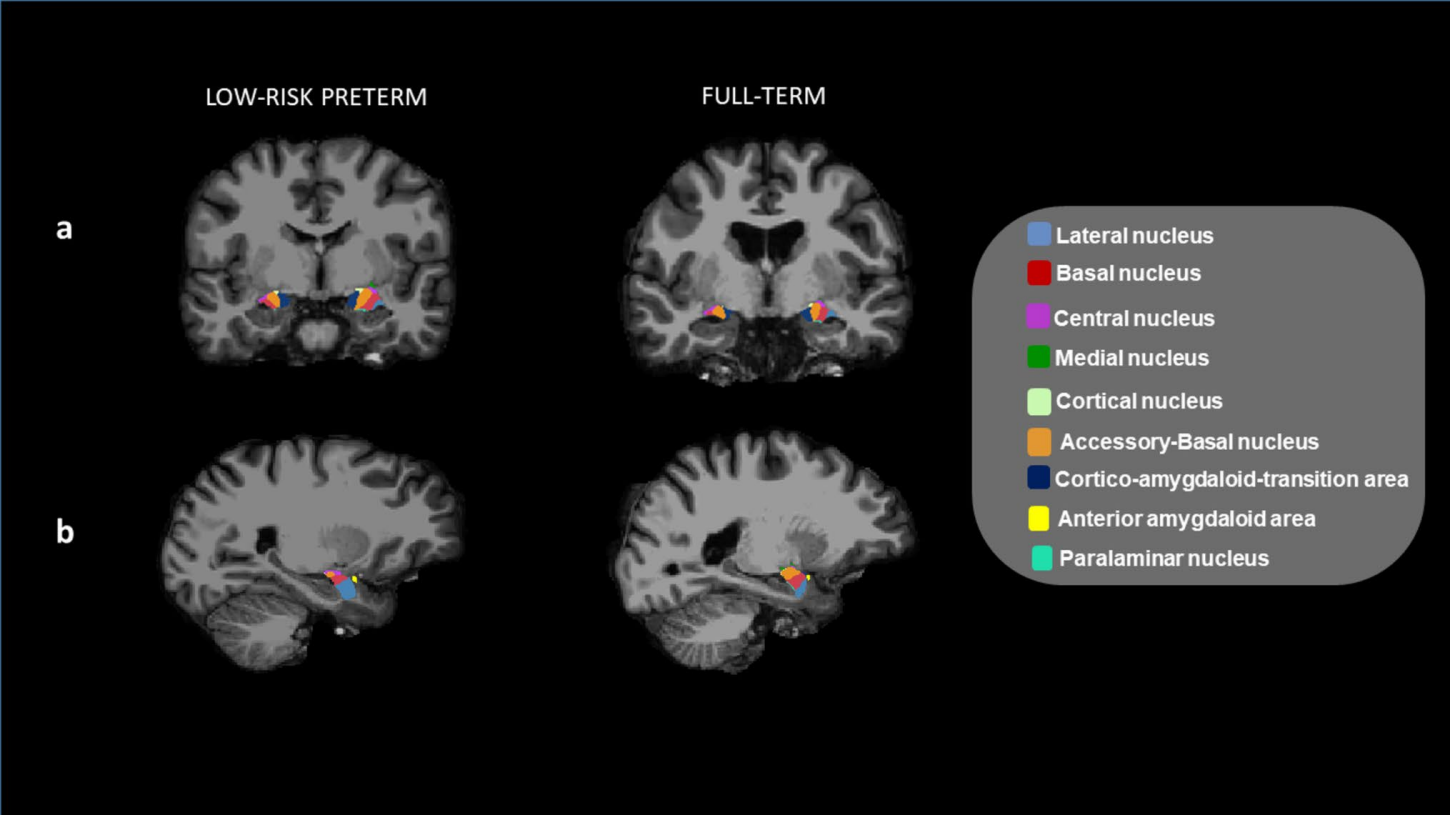
**Right and left subnuclei amygdala volumes**. *Note*: Coronal (**a**) and sagittal (**b**) views showing volumetric segmentation among groups in subnuclei amygdala volumes. In accordance with radiological convention, the left side is shown on the right of the image for each view. Coronal coordinate (**a**): *x*:40.23, *y*:16.33, *z*:−2.30; and sagittal coordinate (**b**): *x*:43.67, *y*:22.13, *z*:−6.46

No significant differences were evidenced in relation to the whole left and right amygdala volumes or amygdala subnuclei volumes between young adults born low-risk preterm and full-term controls (see Table 3, Fig. 1).

**Table 3:** Differences in whole and subnuclei amygdala volumes between low-risk preterm and full-term young adults

	Low-risk preterm *n* = 33 mean volume mm^3^ ± SD	Full-term *n* = 29 mean volume mm^3^ ± SD	*F*-snedecor statistic (*p*)		${\eta }_{p}^{2}$
**Whole amygdala volumes** (adjusted for age, eTIV, and self-SES)					
Left amygdala	1714.71 ± 204.67	1722.45 ± 197.34	0.03 (0.86)		0.00
Right amygdala	1818.53 ± 217.36	1837.92 ± 222.57	0.16 (0.69)		0.00
**Left amygdala segmentation** (adjusted for age, left amygdala volume, and self-SES)					
Lateral nucleus	621.61 ± 72.34	630.50 ± 77.01	1.68 (0.20)		0.03
Basal nucleus	440.44 ± 55.01	438.79 ± 49.69	0.28 (0.60)		0.01
Accessory basal nucleus	264.43 ± 35.39	261.61 ± 33.89	0.69 (0.41)		0.01
Anterior amygdaloid area	53.81 ± 7.91	55.27 ± 7.83	1.21 (0.28)		0.02
Central nucleus	45.05 ± 7.30	43.28 ± 7.84	1.61 (0.21)		0.03
Medial nucleus	26.56 ± 5.95	25.54 ± 6.11	0.55 (0.46)		0.01
Cortical nucleus	27.19 ± 4.17	26.07 ± 3.98	2.32 (0.13)		0.04
Corticoamygdaloid transition	187.59 ± 24.20	186.19 ± 24.20	0.35 (0.56)		0.01
Paralaminar nucleus	51.66 ± 6.29	51.08 ± 6.16	0.40 (0.53)		0.01
**Right amygdala segmentation** (adjusted for age, right amygdala volume, and self-SES)					
Lateral nucleus	668.92 ± 82.36	669.13 ± 85.81	0.00 (0.98)		0.00
Basal nucleus	466.98 ± 58.02	469.45 ± 57.26	0.66 (0.42)		0.01
Accessory basal nucleus	278.64 ± 35.20	279.55 ± 37.33	0.07 (0.79)		0.00
Anterior amygdaloid area	58.90 ± 8.96	59.81 ± 8.81	0.40 (0.53)		0.01
Central nucleus	48.74 ± 8.35	46.84 ± 6.75	1.50 (0.23)		0.03
Medial nucleus	28.04 ± 7.06	26.11 ± 5.03	1.66 (0.20)		0.03
Cortical nucleus	28.74 ± 5.01	27.73 ± 4.57	1.42 (0.24)		0.02
Corticoamygdaloid transition	194.99 ± 22.86	195.55 ± 25.55	0.06 (0.81)		0.00
Paralaminar nucleus	53.66 ± 7.44	53.43 ± 7.06	0.06 (0.81)		0.00

Note: *mm*^*3*^ cubic millimeter, *SD* standard deviation, *eTIV* total intracranial volume, *SES* socioeconomic status, ${\eta }_{p}^{2}$ partial eta squared

Neither were significant differences found between the total score (*F* = 0.97, *p* = 0.33) and seven individual dimensions of resting-state cognition (*F*_DoM_ = 0.11, *p* = 0.74; *F*_ToM_ = 1.89, *p* = 0.17; *F*_Self_ = 2.62, *p* = 0.11; *F*_Plan_ = 0.49, *p* = 0.49; *F*_Sleep_ = 0.04, *p* = 0.85; *F*_Comfort_ = 0.01, *p* = 0.91; *F*_SomA_ = 0.04, *p* = 0.83) between the groups in the ARSQ.

**Fig. 2:**
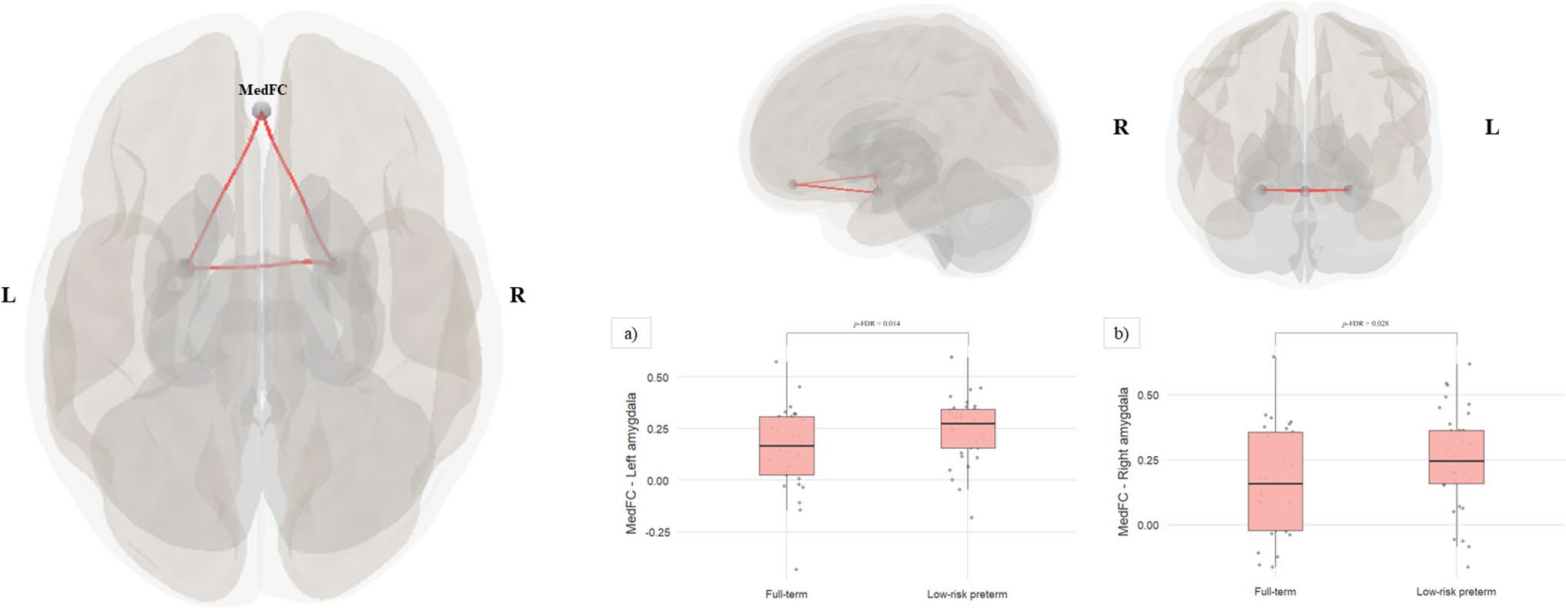
**Left and right amygdala–MedFC FC differences between low-risk preterm and full-term young adults**. *Note*: Low-risk preterm young adults showed left and right amygdala–MedFC FC differences at cluster level compared to the full-term group. The significant results obtained at connection level were **a** increased FC between the left amygdala and MedFC, and **b** increased FC between the right amygdala and MedFC. Results were displayed using CONN Toolbox 21.a and Jamovi 1.6.1

Low-risk preterm young adults evidenced left and right amygdala–MedFC FC differences at cluster level (*F* = 9.89, *p*-FWE = 0.009) compared to the full-term group (see Fig. 2). Specifically, young adults born low-risk preterm exhibited the following: (a) increased FC between the left amygdala and MedFC (*T* (58) = 2.93, *p*-FDR = 0.014), and (b) increased FC between the right amygdala and MedFC (*T* (58) = 2.25, *p*-FDR = 0.028) in comparison to their full-term peers. Nevertheless, no significant FC differences between the left amygdala and right amygdala (*T* (58) = 1.69, *p*-FDR = 0.096) were observed between the groups.

### Aim 3: correlation analysis

No significant correlations were found in the low-risk preterm group or full-term group between social-emotional outcomes and amygdala volumetric values (i.e., whole and subnuclei volumes).

**Fig. 3:**
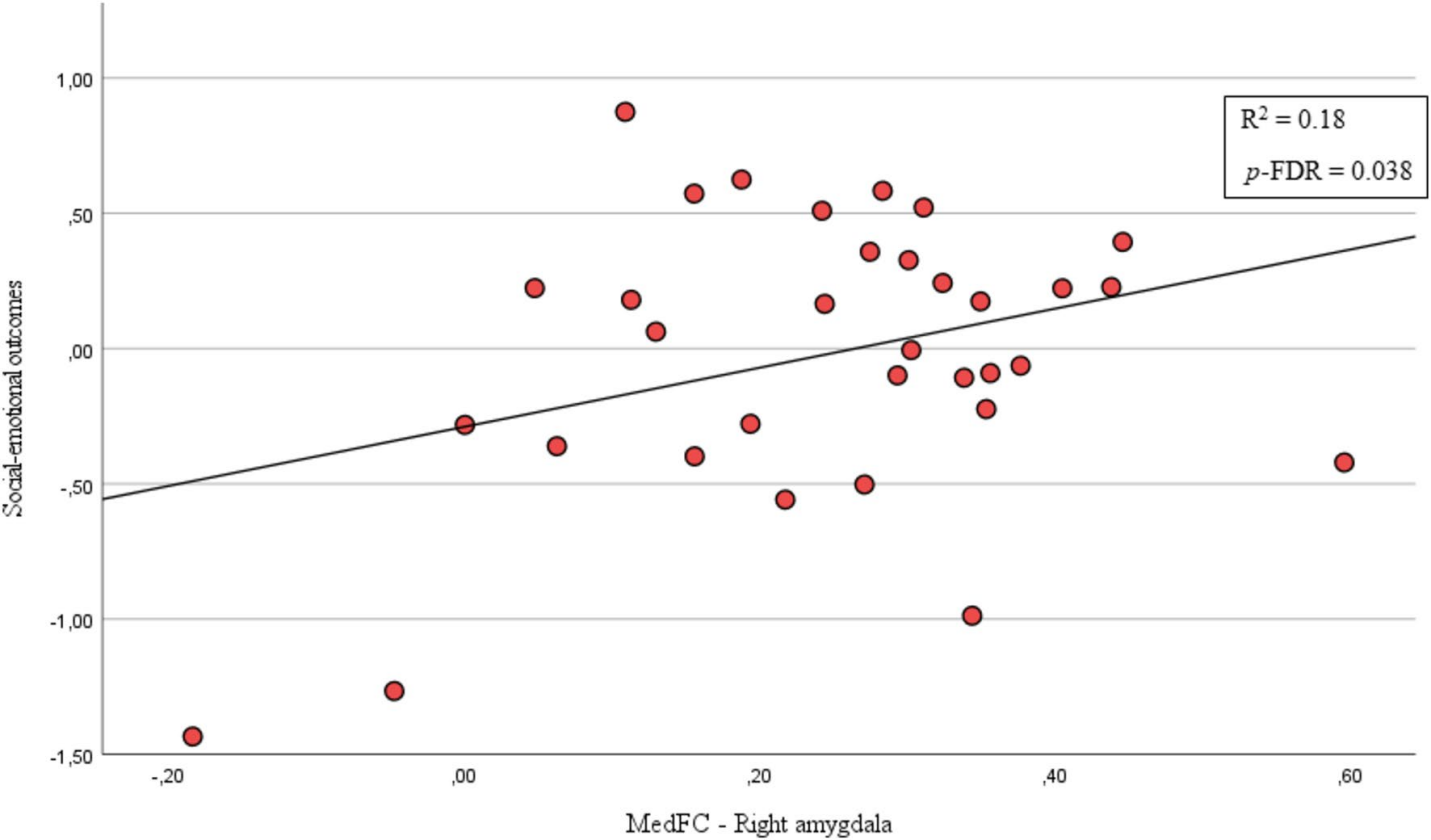
Right amygdala–MedFC FC correlates with social-emotional outcomes in low-risk preterm young adults

Positive correlations (*F* = 6.60, *p*-FWE = 0.036) were found at cluster level between the strength of left and right amygdala–MedFC and social-emotional outcomes only in young adults born low-risk preterm. Specifically, right amygdala–MedFC strength was positively related to social-emotional outcomes (*T* (31) = 2.64, *p*-FDR = 0.038) in low-risk preterm young adults, albeit not in the full-term group. In other words, right amygdala–MedFC increased FC was associated with better social-emotional outcomes in young adults born low-risk preterm (see Fig. 3). However, specific to the low-risk preterm group, left amygdala–MedFC strength did not correlate with social-emotional outcomes (*T* (31) = 1.91, *p*-FDR = 0.064). Moreover, no significant correlations were found between left and right amygdala FC strength and social-emotional outcomes in either group.

## Discussion

To our knowledge, this is the first study that has assessed amygdala volume and its FC in young adults born preterm who are considered to be at lower risk of exhibiting neurodevelopmental alterations. Although low-risk preterm delivery had no effect on social-emotional outcomes or whole and subnuclei amygdala volumes during young adulthood in this sample, increased FC strength between left and right amygdala and MedFC was nonetheless found. The human brain is equipped with a high degree of specialization in processing social inputs, with control ranging from neurotransmitter to neural network level (Dunbar 2009). In other words, an optimal social performance is sustained by a healthy development of the social brain (Galván 2017). According to Kennedy and Adolphs (2012), the best approach to understanding the social brain, including its dysfunction and recovery, is through large-scale networks rather than discrete structures. In fact, similarly, children and adolescents born very preterm also have enhanced FC in and between networks involved in higher-order cognitive performance (Wehrle et al. 2018).

### Social-emotional outcomes

Prematurity has been deemed an independent risk factor with regard to psychiatric disorders; social-emotional problems are particularly prevalent in samples of individuals who were born very preterm (Montagna and Nosarti 2016). For instance, Gilchrist and colleagues (2022) identified a different biological basis for externalizing problems in adolescents born very preterm. Equally, a higher risk of delayed social competence in the first 2 years of life has been associated with births between 32 and 36 weeks’ GA (Cheong et al. 2017; Johnson et al. 2015). However, according to this study covering young adulthood, there is a lack of social-emotional impairment due to prematurity per se rather than other neonatal comorbidities excluded in the sample characterization. Comparable to our results, up to the age of 5 years, no specific preterm social phenotypes have been established (Dean et al. 2021).

### Amygdala structure and FC

Even while amygdala volume is lastingly reduced in heterogeneous preterm samples during both childhood and adulthood (Chau et al. 2019; Schmitz-Koep et al. 2021a), our results displayed no differences in amygdala volumetric values following low-risk preterm birth in current sample. These findings are in line with Rogers’ study targeting children born late preterm (Rogers et al. 2014). Moreover, following very preterm birth, smaller right basal, medial, and cortical nuclei volumes have been linked to social-emotional problems during childhood (Mueller et al. 2022). Meanwhile, increased right centromedial amygdala response to social-emotional events has been displayed in typically developing adolescents (Miller et al. 2021).

Furthermore, with a small GA, both reduced and elevated FC are established between the left amygdala and other areas during the adult stage (Papini et al. 2016). Current findings show that assessed low-risk preterm young adults have an increased FC in the following three structures: left amygdala, right amygdala, and MedFC. In other words, FC was greater between left and right amygdala as well as between left and right amygdala and MedFC at cluster level following low-risk prematurity when compared to adults born full term. Nevertheless, as other studies have suggested, reduced strength between amygdalar and thalamic networks during childhood, as well as reductions in the intrinsic FC between the centromedial subdivision of the right amygdala and prefrontal cortex in young adults, may potentially underlie emotional deficits following very preterm delivery (Mueller et al. 2022; Papini et al. 2014). The results obtained from this study, however, did not support a different FC between left and right amygdala at connection level in these adults born low-risk preterm.

### Amygdala structural and FC correlates with social-emotional outcomes

Our findings reveal no associations between whole or subnuclei amygdala volumes and social-emotional outcomes. Accordingly, no associations between reduced basolateral amygdala volumes and increased social anxiety in adults born very preterm have been found either (Schmitz-Koep et al. 2021b). In healthy developing young adults, in contrast, the experience of stress during the first half of pregnancy may have enduring effects on amygdala structure and may consecutively predict depressive symptoms (Mareckova et al. 2022). Nonetheless, as mentioned above, rather than precise structures, networks may be what sustain social brain activity (Kennedy and Adolphs 2012).

Lastly, this study indicates that both the right amygdala–MedFC and left amygdala–MedFC increased strengths at cluster level were related to social-emotional outcomes in assessed young adults born low-risk preterm. Higher FC between these areas is linked to optimal social-emotional adult functioning following low-risk prematurity. Likewise, better social-emotional abilities have been associated with improved engagement across different networks in children born very preterm (Siffredi et al. 2022). However, increased amygdala–ventral medial prefrontal brain FC has also been related to greater levels of sadness in typically developing infants (Thomas et al. 2019), and less mature social-emotional functioning during adolescence has also been associated with positive frontoamygdala FC (Miller et al. 2021). In other words, negative frontoamygdala FC has been deemed crucial in order to properly handle more demanding contexts concerning social-emotional processes (Callaghan and Tottenham 2016). As per Gee and colleagues (2013), accelerated amygdala–prefrontal development may be considered an ontogenetic adaptation in response to early adversity. Discrepancies found between studies could therefore be explained by different degrees of immaturity or by age-related improvements in emotion regulation caused by changes in activation and FC between prefrontal and amygdala circuits (Silvers et al. 2017).

### Limitations

Unlike previous studies, this study evaluated social-emotional outcomes and amygdala structure and its FC in low-risk preterm and full-term individuals during young adulthood. In other words, unlike previous studies using heterogeneous preterm samples, this study employed neonatal and sociodemographic data to attain comparable groups, thus improving generalizability in terms of results. However, there was no longitudinal follow-up from early childhood to young adulthood so as to determine whether social-emotional outcomes or amygdala structure and its FC were developing commonly or not over time. Another drawback is sample size, which limits the interpretation of these results (i.e., a small power of analysis encourages a type 2 error) as well as the estimation of any differences between sex. Furthermore, although FC between the amygdala and prefrontal cortex is known to be critical for social-emotional functioning (Ochsner et al. 2012), other areas or networks that may be involved (i.e., lateral prefrontal cortex and anterior cingulate cortex, among others) were not examined in this study. Lastly, we found a significant difference in self-SES with the low-risk preterm group obtaining lower scores. However, subsequent analyses were adjusted in the case of this variable. This finding resonates with results from other studies in which prematurity has been previously associated with a lower rate of job success during adulthood (Bilgin et al. 2018; Kroll et al. 2017).

## Conclusions

In conclusion, according to the results obtained from this study, low-risk prematurity has no impact on social-emotional outcomes or amygdala volumes during young adulthood. This may suggest that preterm birth does not imply any atypical amygdalar development, since damage to it impairs the recognition of social emotions (Adolphs et al. 2002). Nonetheless, such differences could be found in larger preterm-born samples, given the sample size of the current study that leads to a small power of analysis incrementing type 2 error. Moreover, whereas social-emotional difficulties have been demonstrated in the first years of life (Cheong et al. 2017; Johnson et al. 2015), an increase in amygdalar FC with MedFC was observed in the studied low-risk preterm sample during adolescence and young adulthood. However, additional research is needed to provide more data concerning brain FC development and its associations with social-emotional outcomes in larger low-risk preterm samples.

## Data Availability

The datasets generated during and/or analyzed during the current study are available from the corresponding author on reasonable request.
